# New Preparation of Morphine

**Published:** 1850-05

**Authors:** 


*New Preparation of Morphine.*—At a meeting of the "Suffolk District Medical Society," Dr. Fisher called attention to a new preparation of morphine, with which he is at present experimenting. He dissolves morph-sulph., 2 grs. in 1 ʒ of chloroform ; ten drops, inhaled by the mouth, in cases of phthisis, will give immediate relief to the harassing cough, and sleep follows, which lasts from an hour to an hour and three-quarters. More largely administered, it checks diarrhœa in phthisis, and in doses of from ten to twenty drops, restrains the action of the bowels in dysentery. —*Boston Medical and Surgical Journal.*

